# Long Term Real-World Outcomes of Trifluridine/Tipiracil in Metastatic Colorectal Cancer—A Single UK Centre Experience

**DOI:** 10.3390/curroncol28030208

**Published:** 2021-06-18

**Authors:** Daniel Tong, Lei Wang, Jeewaka Mendis, Sharadah Essapen

**Affiliations:** 1 St Luke’s Cancer Centre, Royal Surrey NHS Foundation Trust, Egerton Road, Guildford GU2 7XX, UK; dtong@nhs.net (D.T.); l.wang1@nhs.net (L.W.); 2 Surrey Clinical Trials Unit, University of Surrey, Egerton Road, Guildford GU2 7XP, UK; a.mendis@surrey.ac.uk

**Keywords:** Trifluridine-tipiracil, Lonsurf, TAS-102, real-world, colorectal cancer

## Abstract

In the UK, Trifluridine-tipiracil (Lonsurf) is used to treat metastatic colorectal cancer in the third-line setting, after prior exposure to fluoropyrimidine-based regimes. Current data on the real-world use of Lonsurf lack long-term follow-up data. A retrospective evaluation of patients receiving Lonsurf at our Cancer Centre in 2016–2017 was performed, all with a minimum of two-year follow-up. Fifty-six patients were included in the review. The median number of cycles of Lonsurf administered was 3. Median follow-up was 6.0 months, with all patients deceased at the time of analysis. Median progression-free survival (PFS) was 3.2 months, and overall survival (OS) was 5.8 months. The median interval from Lonsurf discontinuation to death was two months, but seven patients received further systemic treatment and median OS gained was 12 months. Lonsurf offered a slightly better PFS but inferior OS to that of the RECOURSE trial, with PFS similar to real-world data previously presented. Interestingly, 12.5% had a PFS > 9 months, and this cohort had primarily left-sided and RAS wild-type disease. A subset received further systemic treatment on Lonsurf discontinuation with good additional OS benefit. Lonsurf may alter the course of disease for a subset of patients, and further treatment on progression can be considered in carefully selected patients.

## 1. Introduction

In the UK, colorectal cancer (CRC) is the fourth most common cancer, and the second commonest cause of cancer deaths [1]. First-line palliative chemotherapy often consists of a fluoropyrimidine (Capecitabine or 5-Fluorouracil), and in patients who are fit and of good performance status, this is usually combined with oxaliplatin or irinotecan. Furthermore, where the tumour does not carry an RAS mutation, the addition of an anti-epidermal growth factor receptor inhibitor (e.g., Cetuximab or Panitumumab) to one of these combinations is recommended to achieve the longest overall survival (OS) [2]. The addition of anti-VEGF therapy or anti-EGFR therapy in the absence of RAS mutations can further improve the benefit from chemotherapy [3].

Trifluridine-tipiracil (Lonsurf) is an oral combination of the antineoplastic nucleoside analogue trifluridine (FTD) and the thymidine phosphorylator inhibitor tipiracil (TPI) [4]. Tipiracil has both a potentiating effect on trifluridine, and a separate anti-angiogenic effect. A phase II study from Japan and the subsequent phase III RECOURSE trial demonstrated the safety and efficacy of Lonsurf in 2014 and 2015, respectively [5,6]. Lonsurf showed efficacy against placebo in the RECOURSE trial, improving overall survival from 5.3 months to 7.1 months, with a hazard ratio (HR) of 0.68 [6]. Progression-free survival (PFS) was improved with a hazard ratio of 0.48. The response rate was only 1.6% but disease control rate, which includes stable disease, was 44%, as compared to 16% with placebo. The commonest adverse event was neutropenia, and there was one treatment-related death. Based on the trial findings, Lonsurf then obtained FDA approval for the treatment of advanced colorectal cancer [7]. These studies led to this drug being made available in Europe and the UK in 2016, in refractory mCRC. In the UK, Lonsurf is the only agent approved by the National Institute for Health and Care Excellence (NICE) for use in patients who have become refractory to fluoropyrimidine, oxaliplatin and irinotecan chemotherapy [8].

With regard to real-world outcomes, PRECONNECT is a large multi-center international Phase IIIb study with 798 patients [9]. It has reported safety, quality of life and PFS results. No OS data are available due to the short follow-up of just 28 days after treatment termination. Median PFS was 2.8 months, an improvement on the results of RECOURSE. Other data available include published retrospective data, meta-analysis and abstracts [10,11,12,13,14,15]. Their reported outcomes are summarised in Table 1.

A limitation of the pre-existing real-world data is the lack of long-term follow-up. We therefore examined the outcomes for patients treated with Lonsurf at a large tertiary Cancer Centre in the southeast of the United Kingdom, with a minimum of two years follow-up. Our study is the largest published review from a single institution in the United Kingdom.

## 2. Materials and Methods

In this retrospective review, data were collected on all metastatic colorectal cancer (mCRC) patients treated with Lonsurf at a tertiary Cancer Centre between 2016 and 2017. Patients without a minimum of two years follow-up, unless due to death, were excluded. Data were collected from electronic patient notes, analysed and stored in a secured Trust network drive. Data collected included baseline demographics and tumour characteristics, treatment details prior to, during and after Lonsurf, and treatment outcomes.

All patients had undergone KRAS and NRAS assessment of their cancer. Assays were performed using the Cobas KRAS Mutation Test Kit (Roche, London, UK) and the NRAS mutation detection kit (Entrogen, Longfield, UK). The assessment for BRAF mutation was performed by next generation sequencing on the Ion Torrent PGM platform (ThermoFisher, Paisley, UK).

### Statistical Analysis

Kaplan–Meier curves, graphical illustrations of the survival function, are produced for both overall survival and progression-free survival using the Lifetest procedure (PROC LIFETEST) in SAS^®^ software (SAS Institute Inc., London, UK). The median survival time is also reported for both overall and progression-free survival.

To evaluate the effect of potential predictors on the survival length, Cox proportional hazard models were fitted, separately for overall survival and progression-free survival, using Phreg procedure (PROC PHREG) in SAS^®^ Software. This model evaluates the effect of age (the dichotomy of below or above 65 years), tumour location (left or right), RAS mutation (wildtype or mutant), metastatic at diagnosis (yes or no), sites of metastasis 3 or more (yes or no), time from diagnosis until treatment with Lonsurf greater than 18 months (yes or no), single organ metastasis (yes or no), lung only metastasis (yes or no), liver only metastasis (yes or no) on the length of survival. Resulting hazard ratios along with 95% confidence intervals and *p*-values of one level of the predictor with respect to a reference level are reported. Statistical significance is evaluated at the conventional cut off level of 5%.

## 3. Results

### 3.1. Patient Demographics and Tumour Characteristics

A total of 56 patients were included in the review. The median age was 61 years (range 37–79) and 59% of patients were male (Table 2). Further, 40 patients (71.4%) had a left-sided tumour, 38 (67.9%) had T3 or T4 disease at diagnosis, whilst only two (3.6%) patients had node-negative disease at diagnosis. Thirty-six (66%) patients had metastatic disease at diagnosis, and the median time to metastasis for the remaining cohort was 11 months (range 2–108). The liver was the first site of metastatic disease in the majority of cases (*n* = 39, 69.6%), and 21 patients had liver-only disease. Twenty-one patients (37.5%) had lung disease, and this was the sole site of metastasis in three patients. Sixteen patients (28.6%) had multiple sites of metastases.

All patients had undergone KRAS and NRAS assessment of their cancer. A RAS mutation was found in 23 (41.4%) patients, with only one BRAF mutation identified. Only 26.8% of tumours were assessed for BRAF mutation in this study as BRAF tests were not part of routine molecular profiling requests during the period when this study cohort received Lonsurf, between 2016 and 2017.

### 3.2. Systemic Treatment Received Prior to Lonsurf

Adjuvant chemotherapy was given in 39.3% of the studied population, with 12 patients (54.5%) receiving Fluorouracil-Oxaliplatin (FOLFOX), two having Capecitabine-Oxaliplatin (CAPOX), and one receiving Fluorouracil-Irinotecan (FOLFIRI) (Table 2). The median number of cycles of adjuvant chemotherapy received was 12 (range 2–14) for FOLFOX and FOLFIRI, and two patients both had eight cycles of CAPOX.

All mCRC patients had a minimum of two lines of systemic treatment for their metastatic disease prior to receiving Lonsurf, with three patients (5.4%) receiving three prior lines of treatment. Median cycles of FOLFOX and FOLFIRI received prior to Lonsurf were 12 (range 4–31) and 12 (3–43), respectively.

### 3.3. Lonsurf Treatment Details

**Figure 1:**
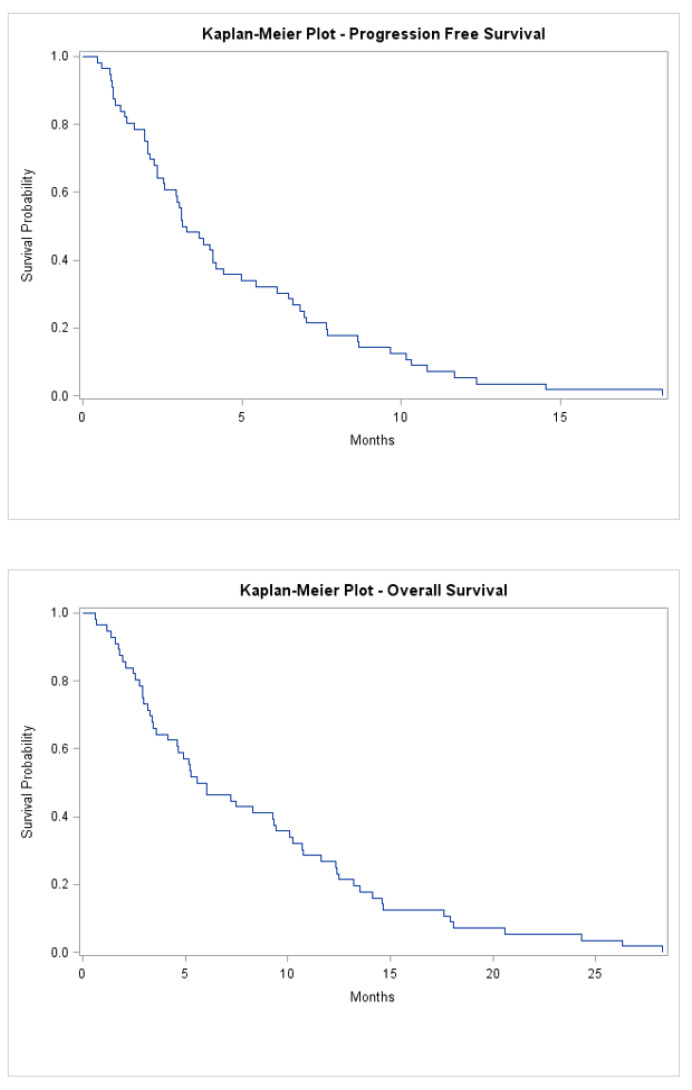
Kaplan-Meier progression-free survival and overall survival of all patients treated.

The median treatment-free interval before starting Lonsurf was 1 month (range 0–23), and the median number of cycles administered was 3 (range 1–16) (Table S1). Median follow-up was 6.0 months (range 1–28), with all patients deceased at time of analysis. The median progression-free survival was 3.2 months (range 1–18), and median overall survival was 5.8 months (1–28) (Figure 1). Seven patients went on to have further lines of systemic treatment; five of whom had Capecitabine monotherapy, while two had FOLFOX. In addition, two patients were referred for consideration of Phase 1 trials.

Among the seven patients who had subsequent therapies, median OS was 12 months (range 1–17) from the point of Lonsurf discontinuation. Four (57.1%) patients had left-sided disease, four had metastatic disease at initial diagnosis, and four had cancers which carried a RAS mutation. The median time to second progression was 7.0 months (range 3–8). Multivariate analysis revealed patients presenting with metastatic disease and aged > 65 at diagnosis had worse outcomes with Lonsurf (Table 3). HR for PFS was 2.7 (95% CI 1.3 to 5.9, *p* = 0.009) among those who were metastatic at diagnosis. In addition, patients aged 65 or greater at the time of diagnosis had worse OS, with HR of 2.1 (95% CI 1.1–4.2, *p* = 0.03).

The reason for Lonsurf discontinuation was progression of disease in 33.3%, general decline in 28.6%, and death within 30 days in 7.1%. Median time from treatment discontinuation to death was two months (range 1–13).

## 4. Discussion

Since the approval of Lonsurf by NICE under National Cancer Drugs Fund (CDF) in 2016, it has become the drug of choice by many as the third line treatment for metastatic CRC. Previous real-world data on Lonsurf have been published, but to our knowledge this is the first published series with a minimum of two years of follow-up data, having followed up every patient until death.

While the median age at diagnosis was 61, the age at which Lonsurf was commenced was 66. The CDF criteria requires a patient to be of PFS 0 or 1 to be eligible for Lonsurf. In this review, twelve patients had PS greater than 1 immediately prior to starting cycle 1. As our cohort includes heavily pre-treated patients with advanced disease, PS may be borderline and prone to fluctuation. There is a 2–3-week delay between chemotherapy referral and the start of treatment, during which patient fitness can further decline. This finding has allowed us to review our cycle 1 pre-assessment protocol in order to flag up changes in PS to the clinical team.

The median PFS for patients with PS greater than 1 prior to cycle one was 3.0 months (range 1–7.8) compared to 3.2 months in the overall study population. Median OS in this subgroup was 3.0 months (range 1–12), compared to 5.8 months (1–28) in the overall group. Patients with deteriorating PS beyond 1 do not appear to benefit significantly from Lonsurf. These results support the recommendation that patients with PS above 1 should not be offered Lonsurf and should be offered best supportive care alone.

Our data is comparable to the results from other investigators summarised in Table 1. The results reported here showed a slightly better median PFS of 3.2 months compared to the 2 months reported in the RECOURSE trial. However, we reported an inferior median OS to that of the RECOURSE trial, namely 5.8 months compared to 7.1 months respectively. The RECOURSE trial was an international study which included patients from across the world, including Japan, USA and the EU and is only one of two prospective studies investigating the benefit of Lonsurf. Though our reported data concern a smaller cohort, their strength lies in the long-term real-world outcomes reported.

Interestingly, seven patients (12.5%) in our series had a good response to Lonsurf, with PFS > 9 months and OS of 13.5 months (range 10–27). We looked for any factors that correlated with better treatment response. Multivariate analysis did not identify factors that were statistically significant apart from those presenting with metastatic disease and aged above 65 at diagnosis. This was however likely due to small numbers underpowering the statistical analysis. Several observations were nonetheless made which may be helpful to be further validated in a larger cohort. For example, all seven patients above had left-sided disease, with four patients having metastatic disease at the time of diagnosis, and only one patient with a RAS mutation. Left-sided tumours are typically less immunogenic and therefore may explain the better response to conventional chemotherapy such as Lonsurf. There is no apparent correlation between sites of metastases and prognosis. However, in this study, three patients had lung-only metastases, and appeared to have a good response to Lonsurf, with median PFS of five months (range 5–9), versus 3.2 months seen in the overall group. Median OS was 12 months (range 9–18) in this lung metastases-only sub-group, versus 5.8 months overall. The numbers are small, and this finding that patients with lung-only metastases may have a better treatment response than those with other sites of metastases has not thus far been reported. One possible explanation for better OS in this subgroup is lower burden of metastatic disease. Lower tumour burden (<3 metastatic sites at trial randomisation) has been reported by Tabernero et al. in their post-hoc exploratory analysis of the RECOURSE trial to be a good prognostic characteristic (GPC) [16]. Patients with lung metastases also appeared to have better PFS and OS response to Lonsurf, and those with lower tumour burden had more favourable response [16]. It is interesting to note that the definition of good prognostic characteristics in this post-hoc analysis also included indolent disease (≥18 months from diagnosis of metastatic disease to trial randomisation) and did not look at lung-only metastases. Nevertheless, these are important observations that may help with selecting patients that would most benefit from Lonsurf.

After treatment discontinuation, the patients studied had a short time to death overall. There was however a subgroup that were able to receive further systemic treatment, and this subgroup had a good median OS of 12 months (range 1–17). There is no clear factor identified associated with a good response to further treatment. In addition, it is difficult to ascertain whether the additional OS gained is due to further treatment received, patient selection, or tumour biology. It would be interesting to look at whether this subgroup shares the same characteristics of the GPC subgroup discussed above.

This is the largest published real-world data from a single institution in the UK with a minimum of two-year follow-up. While Lonsurf offers a small PFS and OS benefit overall, we identified a cohort of patients who experienced a long period of disease control. All of these patients had left-sided disease, and most were RAS wild-type. In addition, a number of patients did go on to receive further systemic therapy after discontinuing Lonsurf, with good effect. It is not clear currently what baseline demographics or tumour characteristics confer this advantage, but this may be due to self-selection of patients with more indolent disease and better fitness.

There is growing evidence, both in recent publications and ongoing trials that may expand the role of Lonsurf in metastatic colorectal cancer. In the third-line setting, Pfeiffer et al. have demonstrated a two-month improvement of median PFS (2.6 vs. 4.6 months) by adding Bevacizumab to Lonsurf in the third-line setting [17]. While this is a Phase II study, the results of an ongoing Phase III study are eagerly anticipated. Similar efficacy was reported by Yoshida et al. in their single-arm Phase II study [18]. While the current role of Lonsurf is as monotherapy, this may change if studies are able to demonstrate increased efficacy when combined with other agents such as Bevacizumab. Importantly, the improvement in OS will be a key consideration by funding agencies. Its role in earlier lines of treatment remains to be answered by ongoing trials [19,20,21]. Our study suggests some tumour-factors that may help select patients who would most benefit from Lonsurf. For instance, with our observation that left-sided tumours demonstrated a better response to Lonsurf, it would be interesting to compare the response between left and right-sided tumours with the addition of Bevacizumab. The ongoing Phase III study may help further clarify the question of patient selection. 

## 5. Conclusions

In summary, Lonsurf is an option for patients progressing beyond second line treatment. In this study, we found that patients with left-sided disease and RAS wild-type had a longer PFS. Although the numbers are small, patients with lung-only metastasis had better PFS and OS. Multivariate analysis was underpowered to confirm these findings and these factors would need to be further validated in a larger cohort. After progression, 12.5% received further fluoropyrimidine-based treatment, suggesting that this is an option for selected patients. Patient selection is clearly key to identifying those who will best benefit from this third-line treatment.

## Figures and Tables

**Table 1:** A summary of pre-existing Lonsurf real-world outcomes compared with trial data.

Study [Reference]	Country	Study Type	Number of Patients	Performance Status	RAS Mutant (%)	Previous Lines of Treatment	Median Duration of Treatment (months)	Median PFS (months)	Median OS (months)
RECOURSE [6]	International	Phase III trial	800	0–1	51	≥2	1.5	2	7.1
PRECONNECT [9]	International	Phase IIIb trial	798	0–1 in 97%	52.6	36.1% ≤2 30.6% 3 32.8% ≥4	2.8	3	Not reported
Stavraka et al. [10]	UK	Abstract (retrospective)	236	0–1 in 90%	Not reported	≥2	3	3.3	7.6
Tilby et al. [11]	UK	Abstract (retrospective)	91	0–1 in 96%	53	Not reported	4.3	4.1	8.7
Samawi et al. [12]	Canada	Retrospective study	717	Not stated	60	Not reported	2.5	Not reported	Not reported
Anderson et al. [13]	Japan, Europe	Meta-analysis	1008	0–1 in 93%	62	34% ≤2 35% 3 31% ≥4	Pooled data not reported, individual studies ranged 1–3	2.2	6.6
Wallander et al. [14]	Sweden	Retrospective study	48	0–1 in 94%	60	73% ≤2 23% 3 2% ≥4	Not reported	2.3	6.4
Cremolini et al. [15]	Italy	Retrospective study	341	0–1 in 98%	59	33.4% ≤2 28.1% 3 38.4% ≥4	Not reported	2.4	6.2

PFS (progression-free survival), OS (overall survival).

**Table 2:** Patient and treatment demographics prior to Lonsurf.

Variable	Value (*n*)	(%)
Baseline characteristics		
Gender		
Male	33	59
Female	23	41
Median age at diagnosis	61	Range 37–79
Location of tumour		
Left-sided	40	71.4
Right-sided	15	26.8
Other	1	1.8
Staging at diagnosis		
T		
1	0	0
2	2	3.6
3	22	39.3
4	16	28.6
N		
0	2	3.6
1	13	23.2
2	25	44.6
M		
0	16	30.8
1	36	69.2
Median interval until metastasis (months)	11	Range 2–108
Site of metastasis		
Lung	21	37.5
Liver	39	69.6
Peritoneum	8	14.2
Nodal	8	14.2
Ovary	2	3.6
Multiple sites of metastases?		
Yes	16	28.6
No	40	71.4
Histology		
Median number of positive nodes	4	Range 0–18
Grade of tumour		
1	0	0
2	32	57.1
3	6	10.7
Not stated or not operated	18	32.1
Extramural vascular invasion		
Positive	16	28.6
Negative	15	26.8
Not stated	25	44.6
RAS mutation		
Mutant	23	41.4
Wildtype	25	44.6
Not tested	8	14.2
BRAF mutation		
Mutant	1	1.8
Wildtype	14	25
Not tested	41	73.2
Systemic treatment details		
Adjuvant chemo received		
Yes	22	39.3
No	34	60.7
Regimen		
FOLFOX	12	54.5
FOLFIRI	1	1.8
CAPOX	2	3.6
5′FU	1	1.8
Capecitabine	1	1.8
Not stated	5	8.9
Median number of cycles received	12	Range 2–14
Lines received prior to LONSURF		
1	0	0
2	52	92.9
3	3	5.4
Not stated	1	1.8
Median FOLFOX received	12	Range 4–31
Median FOLFIRI received	12	Range 3–43

FOLFOX (Fluorouracil-Oxaliplatin), FOLFIRI (Fluorouracil-Irinotecan), LONSURF (Trifluridine-tipiracil).

**Table 3:** Multivariable analysis of the effect of parameters on PFS and OS.

Parameters	PFS		OS	
	HR (95% CI)	*p*-Value	HR (95% CI)	*p*-Value
Age at diagnosis (≥65 vs. <65)	0.9 (0.4–1.7)	0.78	2.1 (1.1–4.2)	0.03
Tumour location (right vs. left)	1.5 (0.6–3.7)	0.43	0.9 (0.4–2.3)	0.86
Metastatic at diagnosis (yes vs. no)	2.7 (1.3–5.9)	0.009	2 (1–4)	0.05
RAS status (wildtype vs. mutant)	0.7 (0.3–1.4)	0.31	1.7 (0.8–3.4)	0.14
Lung only metastasis (yes vs. no)	0.8 (0.2–3.1)	0.81	0.7 (0.2–2.6)	0.64

PFS (progression-free survival), OS (overall survival), HR (hazard ratio).

## Data Availability

The data presented in this study are available on request from the corresponding author. The data are not publicly available due to privacy and ethical reasons.

## Supplementary Materials

The following are available online at https://www.mdpi.com/article/10.3390/curroncol28030208/s1, Table S1. Details of Lonsurf treatment.
