# Supplementary material for: Long Term Real-World Outcomes of Trifluridine/Tipiracil in Metastatic Colorectal Cancer—A Single UK Centre Experience
Source: Curr Oncol. 2021 Jun 18;28(3):2260–9. doi: 10.3390/curroncol28030208 (PMC8293218; doi:10.3390/curroncol28030208)
Supplement: Supplementary file 1 [file curroncol-28-00208-s001.zip › curroncol-1180417-supplementary.pdf]

Supplementary Materials

Table S1. Details of Lonsurf treatment.

**Variable**	**Value**	
	(n)	(%)
Median age commencing Lonsurf	66	Range 34–82
Performance status prior to cycle 1		
0	7	12.5
1	35	62.5
2	11	19.6
3	1	1.8
4	0	0
Not stated	2	3.6
Median interval between last cycle of chemo & cycle 1 Lonsurf (months)	1	Range 0–23
Median number of cycles administered	3	Range 1–16
Interval between treatment discontinuation and death	2	Range 1–13
Reason for discontinuation		
PD	19	33.9
Worsening PS	2	3.6
General decline	16	28.6
Toxicity	1	1.8
Death	4	7.1
Patient preference	1	1.8
Not stated	13	23.2
